# Analyzing the Relationship Between Organizational Politics, Emotional Intelligence, and Employee Behavior: A PLS-SEM Modeling

**DOI:** 10.12688/f1000research.151721.2

**Published:** 2025-04-07

**Authors:** Sonakshi Bhatia, Babita Rawat, Dhani Shanker Chaubey, Farman Ali

**Affiliations:** 1 Management, Uttaranchal University, Dehradun, Uttarakhand, 248001, India

**Keywords:** Organizational politics, emotional intelligence, employee behavior, Behavioral Dynamics, Relationship Conflict, Resource Constraints, Role Conflict.

## Abstract

**Background:**

This study explores the complex relationship between organizational politics, emotional intelligence and employee behavior in contemporary settings. Organizational politics, which is widespread in organizational settings, has a substantial impact on different aspects of employee behavior. Emotional Intelligence has become a vital factor in individuals’ capacity to successfully traverse the complexities of an organization. This study consolidates current material to offer insights into the impact of organizational politics on employee behavior and the mediating role of emotional intelligence in this connection.

**Methods:**

A survey was undertaken with a sample size of 500 employees hailing from several Information Technology (IT) and Information Technology Enabled Services (ITES) firms in the Dehradun (Uttarakhand) region. The study employed quantitative methodologies to investigate the correlations between Emotional Intelligence, Perceived Organizational Politics and Employee Behavior. The data were evaluated using Partial Least Squares Structural Equation Modelling (PLS-SEM) to examine the hypothesized correlations and mediation effects.

**Results:**

The study identified substantial correlations between Emotional Intelligence, Perceived Organizational Politics, and Employee Behavior. Emotional Intelligence was discovered to have a positive impact on Behavioral Dynamics. Perceived Organizational Politics had a substantial influence on both Emotional Intelligence and Behavioral Dynamics. Factors such as Need for Power, Relationship Conflict, Resource Constraints, Role Conflict, and Workforce Diversity were discovered to have an impact on Perceived Organizational Politics.

**Conclusions:**

The results confirm strong connections between organizational politics, emotional intelligence, and employee behavior, highlighting the significance of these concepts in comprehending employee behavior in organizational environments. The study proposes that enhancing employees' emotional intelligence can alleviate the adverse effects of organizational politics, resulting in enhanced behavioral dynamics. The study also addresses the limits, outlines potential areas for further research, and highlights the managerial implications. It suggests that firms should prioritize the development of emotional intelligence to cultivate a favorable work environment.

## Introduction

In an unstable and complicated environment, an organisation is made up of individuals who manage and organise other resources to achieve established goals. Political behavior becomes prevalent and objective because of people’s involvement and the organization’s failure to match their expectations in terms of logic and objectivity. During the 1990s, the concept of organisational politics and workplace perceptions of organisational politics expanded and they are considered an essential component of contemporary business practices. Organizational politics and its impact on employee behavior have garnered significant attention from scholars and practitioners alike. Organizational politics is defined as the pursuit of self-interest within an organization, frequently at the expense of others' legitimate interests. It can take many forms including favoritism, manipulation and coalition-building. Organizational politics can have a significant impact on employee attitudes, job satisfaction and performance ( G. R. Ferris *et al.*, 2013). In recent years, scholars have increasingly acknowledged the importance of emotional intelligence in moulding people's reactions to organizational politics. Emotional intelligence, as defined by ( Salovey & Mayer, 1990) is the ability to notice, comprehend, control, and express emotions effectively. Individuals with high levels of emotional intelligence can negotiate complex social contexts, regulate their emotional responses, and empathize with others' points of view ( Goleman, 1995). This paper will investigate the relationship between organizational politics, emotional intelligence, and employee behavior, relying on previous research to give theoretical insights and practical consequences for firms looking to encourage healthy workplace dynamics.

This study investigates the interplay of organizational politics (OP) and emotional intelligence (EI) on employee behavior, specifically within the IT/ITES sector in North India, hence addressing deficiencies in current research. This research examines the interaction between OP and EI, focusing on the mediating and moderating functions of EI within the OP-behavior relationship, in contrast to previous studies that have mostly analyzed them separately. Asad *et al.* (2014), focused only on mediating role of Emotional Intelligence and Melese Ambaw *et al.* (2021) and Yaseen (2020) focused on moderating role only. Insufficient attention has been devoted to the moderating impact of emotional intelligence, with the majority of available research focusing on areas such as healthcare, education, and banking, or being done in Western or non-North Indian contexts. This study conducts a thorough empirical investigation of the IT/ITES sector in North India, generating significant findings for academia and industry.

## Review of related literature

### Factors influencing organization politics

**Relationship conflict:**

Relationship conflict inside organizations has been extensively researched as a predictor of organizational politics. ( Jehn, 1995) discovered that relational conflict, defined as interpersonal tension and hatred, strongly contributes to the establishment of political behaviors inside teams and departments. Such disputes are frequently caused by differences in employee personalities, values, or communication styles, which result in power struggles and manipulative activities aimed at securing personal interests above organizational goals.

If unresolved, these conflicts promote political behaviors—actions intended for personal gain—resulting in an environment of distrust and detrimental competitiveness (Sahoo & Sahoo, 2019). Efficient dispute resolution, supported by a transparent and equitable organizational culture, is essential for sustaining a healthy workplace atmosphere (Sahoo & Sahoo, 2019). Leaders that advocate for equity and openness diminish the probability of political maneuvering, but authoritarian or laissez-faire leadership styles may intensify conflicts ( Chen *et al.*, 2019). Emotional intelligence (EI) and conflict management skills are essential in mitigating the negative impacts of relational conflict. Chen *et al.* (2019) discovered that managers possessing elevated emotional intelligence navigate conflicts more adeptly, resulting in improved conflict resolution and a more favorable workplace environment. Moreover, Vapiwala and Pandita (2025) contend that cultivating an organizational learning culture augments employees’ emotional intelligence, resulting in enhanced conflict resolution abilities ( Vapiwala & Pandita, 2025).

These reasons give rise to the following hypothesis:

H1: Relationship Conflict contribute significantly towards organizational politics

**Role conflict:**

Role conflict, which results from differences or inconsistencies in work expectations, has been established as a strong predictor of organizational politics ( Rizzo *et al.*, 1970). When employees encounter opposing demands from numerous sources, like as bosses, coworkers, or organizational regulations, they may use political methods to manage the tensions and preserve their own interests. This dynamic hamper workflow while also undermining trust and collaboration inside the business, creating an environment amenable to political manipulation.

Augmented Communication Constructive conflict resolution promotes transparent and copen communication. By confronting difficulties directly and respectfully, employees acquire the ability to articulate their perspectives clearly and attentively consider others, so cultivating a culture of transparency and mutual respect ( Bagga *et al.*, 2023). Enhanced Relationships Addressing disagreements constructively fosters trust and enhances relationships among employees. Effective conflict resolution fosters enhanced understanding and collaboration, resulting in a more unified and supportive workplace.

These reasons give rise to the following hypothesis:

H2: Role Conflict contribute significantly towards organizational politics

### Resource constraints

Resource constraints, such as limited funds, time, or people, have been associated to increased organizational politics ( Cropanzano *et al.*, 2017). When resources are tight, competition among personnel and departments heats up, resulting in strategic manipulation and coalition-building to get access to critical resources. Furthermore, resource scarcity exacerbates tensions and conflicts by forcing individuals to compete for a bigger share of limited resources, maintaining a culture of political gamesmanship and opportunism inside the organization.

Pindek and Spector (2016) discovered that organizational restrictions, including insufficient resources, adversely influence employee motivation, therefore affecting performance. Perceived inequity in resource allocation may diminish motivation, leading employees to resort to political behaviors as coping strategies. The Job needs-Resources (JD-R) paradigm asserts that a disparity between job needs and available resources results in strain and stress for employees. This strain may result in counterproductive work practices, including political acts, as employees strive to manage the challenges of resource scarcity. The JD-R model emphasizes that job resources, such as social support and a favorable work environment, can mitigate the adverse impacts of job demands, hence decreasing the probability of political behaviors stemming from resource limitations.

These reasons give rise to the following hypothesis:

H3: Resource constraints Conflict contribute significantly towards organizational politics

### Need for power

Individual variations in the desire for power have been identified as crucial determinants in organizational politics ( Winter, 1973). Employees that have a strong desire for power are more prone to participate in political activities in order to exert authority, control information and influence decision-making processes inside the firm. Their activities frequently favor personal ambition over communal goals, causing increasing friction and distrust among coworkers. As a result, the presence of individuals with a high desire for power can aggravate political dynamics while undermining organizational cohesiveness and performance.

Khattak *et al.* (2023) investigated the moderating effect of servant leadership on the association between perceived organizational politics and employee performance results. Their findings suggest that servant leadership mitigates the adverse impacts of organizational politics on employee task performance and organizational citizenship behaviors. This leadership style, which emphasizes employee welfare and growth, cultivates a supportive atmosphere that mitigates the effects of organizational politics. Mosquera *et al.* (2024) examined the impact of ethical leadership on meaningful work and turnover intentions, while accounting for the mediating effect of perceived organizational politics. The research indicated that ethical leadership diminishes views of organizational politics, enriches the sense of meaningful work, and lowers turnover intentions. By advocating for ethical norms and clear communication, ethical leaders can alleviate the adverse effects of organizational politics.

These reasons give rise to the following hypothesis:

H4: Power motive among employee contribute significantly towards organizational politics

### Workforce diversity

Workforce diversity, which includes disparities in demographics, histories, and opinions, has been shown to influence the terrain of organizational politics ( Shore *et al.*, 2009). While diversity promotes innovation and creativity, it may also lead to disputes caused by cultural misunderstandings, prejudices, and power disparities. In diverse work contexts, individuals may use political methods to negotiate complicated social dynamics, defend their interests, or push their agendas, worsening internal tensions and divides. As a result, good diversity management is critical for limiting the harmful influence of organizational politics while also building inclusive and egalitarian workplace environments.

The interplay between workforce diversity and organizational politics is intricate and multifaceted. Diverse workforces contribute to a range of perspectives and foster innovative problem-solving methods. Nonetheless, they can lead to misunderstandings, conflicts, and perceptions of favouritism, thereby promoting organizational politics. Mishra *et al.* (2016) conducted a study at a central university in India, demonstrating that workforce diversity significantly influences perceptions of organizational politics. The research indicated that challenges related to diversity may result in a greater likelihood of employees intending to leave the organization, increased job-related anxiety, and diminished organizational commitment. Joep Hofhuis and Otten (2016) conducted a study demonstrating that a positive diversity climate fosters trust and openness in workgroup communication, which in turn enhances work outcomes.

These reasons give rise to the following hypothesis.

H5: Workforce diversity among employee contribute significantly towards organizational politics

### Organizational politics and employee behavior

Organizational politics is a complicated phenomenon that is inextricably linked to employee behavior, providing a fertile ground for research in organizational psychology and management. Scholars have intensively researched the multidimensional character of organizational politics, emphasizing its ubiquitous presence in workplace dynamics ( Gotsis & Kortezi, 2010). Studies have shown that it has an impact on several aspects of employee behavior, including work satisfaction, organizational commitment, and performance ( Ng & Feldman, 2012; Hochwarter *et al.*, 2020). Furthermore, recent research has focused on the methods by which organizational politics impact employee attitudes and behaviors, highlighting the importance of perceptions, power dynamics, and organizational culture ( Hochwarter *et al*., 2020). In addition, emerging trends in the literature have investigated the intersection of organizational politics with present-day problems such as remote work arrangements and digital communication platforms, throwing light on new forms and manifestations of political behavior in modern organizational contexts. The literature on organizational politics and employee behavior reflects a nuanced understanding of the intricate interplay between organizational structures, individual motivations, and workplace interactions, offering valuable insights for practitioners and scholars alike. Organizational politics can exert a significant influence on various dimensions of employee behavior, including job satisfaction, organizational commitment, and job performance. Research indicates that perceptions of organizational politics are negatively correlated with employee job satisfaction and commitment ( Kacmar & Ferris, 1991). Employees who perceive their organizations as politically charged may experience heightened levels of stress, reduced job satisfaction, and increased turnover intentions ( Vigoda-Gadot, 2006). Moreover, organizational politics can shape employees' behavioral responses, leading to detrimental outcomes such as increased absenteeism, decreased organizational citizenship behaviors, and lower job performance ( Drory & Romm, 1990). Employees may engage in defensive behaviors, such as withholding effort or information, to protect themselves from the perceived negative consequences of organizational politics ( Kacmar & Ferris, 1991).

These arguments leads to the following hypothesis:

H6: Organisational politics have significant effect on employee behavior

### Organisational politics and emotional intelligence

Organizational politics has received substantial attention in the literature due to its complex influence on personnel and organizational results. Scholars have increasingly acknowledged the complex link between corporate politics, emotional intelligence (EI) and employee behavior dynamics. Liu *et al.* (2021), found that exposure to organizational politics can have a considerable impact on people's emotional intelligence, specifically their capacity to identify, comprehend, and control emotions successfully. This conclusion is confirmed by a research by Drory and Meisler (2016), which focuses on the negative impacts of organizational politics on employees' emotional intelligence, resulting in worse job satisfaction and higher turnover intentions. Naseer *et al.* (2016) for example, found that persons with higher emotional intelligence may be better equipped to navigate and cope with organizational politics, mitigating its negative impact on job performance and well-being. However, the link between organizational politics and emotional intelligence is multifaceted and may differ depending on contextual factors such as corporate culture and leadership style ( Ghosh & Ghosh, 2019). Overall, the literature emphasizes the necessity of addressing organizational politics as a major element impacting employees' emotional intelligence and, by extension, organizational performance. Emotional intelligence is critical in balancing the link between corporate politics and employee behavior. Individuals with high levels of emotional intelligence are better able to deal with the emotional demands of organizational politics and employ constructive coping techniques ( Goleman, 1995). They are better at managing their own emotions, empathizing with others' viewpoints, and resolving problems peacefully ( Salovey & Mayer, 1990). Empirical investigations have shown that emotional intelligence buffers the negative consequences of organizational politics on employee attitudes and behaviors ( Abraham, 2000). For example, employees with high emotional intelligence may be less likely to perceive organizational politics as threatening, thus reducing their propensity to engage in defensive behaviors or experience negative emotional outcomes ( Jordan & Troth, 2002).

Goleman *et al.* (2013) found that emotional intelligence plays an important role in promoting favorable workplace outcomes such as job satisfaction, organizational commitment, and successful leadership. Furthermore, research by Brackett & Salovey (2006) emphasizes the importance of EI in influencing employees’ interpersonal relationships, conflict resolution abilities, and adaptation to changing work contexts. Furthermore, Bar-On (2010) findings indicate the influence of EI on lowering workplace stress and improving employee mental well-being, which contributes to overall organizational productivity and performance. However, inconsistent data exist about the magnitude of EI’s effect on various behavioral outcomes, emphasizing a need for more empirical research ( Mayer & Salovey, 2007). Thus, while existing literature emphasizes the relevance of EI in affecting employee behavior, future research should dive deeper into the processes by which EI functions and the consequences for varied organizational contexts. Furthermore, EI has been connected to decreased turnover intentions and higher work satisfaction ( Miao *et al.*, 2017). Zeidner *et al.* (2016) conducted a study that underlined the relevance of emotional intelligence in leadership, demonstrating its influence on team dynamics and organizational environment. However, subsequent research have highlighted significant limits, such as the cultural and environmental uniqueness of EI components ( Miao *et al*., 2021).

Previous study has shown that organizational politics, which are characterized by power conflicts, favoritism, and manipulation, have a major influence on employee attitudes, work satisfaction, and performance ( Hochwarter *et al.*, 2020). Concurrently, EI, defined as the ability to sense, comprehend, and successfully control emotions, has emerged as a critical component in determining individual achievement and organizational effectiveness ( Mayer & Salovey, 2007). Recent research has highlighted the mediation function of EI in moderating the harmful impacts of organizational politics on employee behavior ( Turi & Sarfraz, 2023). Employees with high EI, for example, may be able to better manage political circumstances, regulate their emotions in reaction to organizational pressures, and maintain healthy interpersonal connections despite political problems ( Blaik-Hourani *et al*., 2023). Furthermore, emotional intelligence has been associated to improved conflict resolution abilities, resilience, and adaptive coping mechanisms, all of which lead to more constructive responses to organizational politics ( Jordan & Troth, 2002). Understanding the relationship between organizational politics, EI, and employee behavior is critical for firms seeking to develop a healthy work environment and improve employee well-being and performance.

These arguments lead to the assumption of following hypothesis:

H7: Emotional Intelligence of employee have positive and significant effect on Employee Behavior

H8: Emotional Intelligence of employee mediates the relationship between organisational politics and employee behavior

### Theoretical framework

The purpose of this study is to analyze the complex relationship between organizational politics, emotional intelligence (EI), and employee behavior using Partial Least Squares Structural Equation Modeling (PLS-SEM). Organizational politics, defined as the use of power and influence strategies to achieve personal or organizational objectives, has received a lot of attention in organizational behavior research because of its widespread impact on workplace dynamics ( Mintzberg, 1985; D. L. Ferris *et al.*, 2008). Emotional intelligence, on the other hand, refers to the capacity to identify, analyze, and control one's own and others' emotions, which has been identified as a critical predictor of occupational performance and interpersonal interactions ( Goleman, 1995). This research proposes a theoretical framework based on current literature in both organizational politics and emotional intelligence, which may act as a moderator in the interaction between organizational politics and employee behavior. Using PLS-SEM, this study aims to provide a nuanced understanding of how emotional intelligence may mitigate or exacerbate the effects of organizational politics on employee behavior, thereby providing valuable insights for both scholars and practitioners in managing organizational dynamics effectively.

## Methods

The relationship between organizational politics, emotional intelligence, and employee behavior is investigated quantitatively in this study using Partial Least Squares Structural Equation Modeling (PLS-SEM). The PLS-SEM approach was chosen because it is ideal for investigating complicated interactions in theoretical models with limited sample sets ( Sarstedt *et al.*, 2022). This section describes the research strategy, sample approach, data collecting tools, and analytical methods used in this study. A cross-sectional study strategy is used to collect data from participants from multiple organizations in November 2023. This method enables the investigation of connections between variables without the necessity for longitudinal data collection ( Sarstedt *et al*., 2022). The research uses convenience sampling to identify individuals from a variety of sectors and organizational contexts. The research uses convenience sampling to identify individuals from a variety of sectors and organizational contexts. A sample size of at least 500 employees is intended to achieve acceptable statistical power and representativeness ( Sarstedt *et al.*, 2022). Participants were recruited using internet platforms and professional networks, assuring broad geographical coverage and diverse demographic representation.

The study utilized validated scales to measure key constructs. Organizational politics was assessed using a six-item scale from the original 15-item **Perception of Organizational Politics Scale (POPS)** by Kacmar and Ferris (1991). Antecedents of organizational politics were measured, as follows: Resource Scarcity was evaluated using a four-item scale adapted from Pareek (1983b) original eight-item scale; Workforce Diversity perception was assessed with three items from Jehn *et al.* (1999) six-item scale; Role Conflict was measured using nine items from Rizzo *et al.* (1970) original 15-item scale; Relationship Conflict was measured through a five-items adapted from Jehn (1995) eight-item scale; 12-item scale developed by Pareek (1983a), was used to assess employees’ Need for Power, the Wong and Law Emotional Intelligence Scale (WLEIS) ( Wong & Law, 2017), and the Employee Behavior Inventory ( Spector & Fox, 2002). The Organizational Politics Scale measures employees’ perceptions of organizational politics, including favoritism, influence, and networking. The WLEIS assesses employees’ emotional intelligence across four dimensions, while the Employee Behavior Inventory evaluates various dimensions of employee behavior.

Partial Least Squares Structural Equation Modeling (PLS-SEM) is used to analyze the complex relationships between organizational politics, emotional intelligence, and employee behavior. PLS-SEM is a robust technique for modeling complex theoretical frameworks with latent variables and observed indicators, making it suitable for exploratory research. The analysis involves two stages: measurement model assessment and structural model evaluation. In the measurement model assessment stage, the instruments' reliability and validity are evaluated through internal consistency, convergent validity, and discriminant validity. The structural model is estimated to examine relationships between latent constructs and test hypothesized paths. Bootstrapping procedures are used to assess the significance of direct and indirect effects in the model. Table 1 indicates the demographic characteristic of employees.

**Table 1.** Demographic Characteristics of Respondents.

Category	Description	Frequency	Percent
Age category	upto 25 years	52	10.4
	26years to 35years	87	17.4
	36years to 45years	148	29.6
	46 years to 55years	128	25.6
	56years and above	85	17.0
Gender Category	Male	269	53.8
	Female	231	46.2
Marital Status	Married	202	40.4
	Unmarried	298	59.6
Education Level	Graduation	31	6.2
	Post Graduation	6	1.2
	Professional Qualification	113	22.6
	PhD	321	64.2
	Others	29	5.8
Income Level	Rs20001 to Rs.30000 PM	9	1.8
	Rs.30001 to Rs.40,000 PM	13	2.6
	Rs.40, 001 To Rs50,000PM	175	35.0
	Rs 50001 to 60,000PM	133	26.6
	Rs 60,001and Above	170	34.0
Years of Experience	0-1 Years	51	10.2
	2-5 Years	215	43.0
	6-10 Years	164	32.8
	More than 10 years	70	14.0

Source: Author’s Compilation.

## Results

Table 1 shows the demographics of respondents to research on the link between politics, emotional intelligence, and employee behavior. In terms of age distribution, the majority of respondents are between the ages of 36 and 45 (29.6%) and 46 to 55 (25.6%). This suggests a high representation of middle-aged people in the research. However, it is worth mentioning that respondents aged 26 to 35 years (17.4%) and 56 years and older (17.0%) make significant contributions to the sample. In terms of gender, the sample looks to be reasonably balanced, with slightly more males (53.8%) than females (46.2%). In terms of marital status, the majority of respondents are single (59.6%), with a sizable proportion married (40.4%). This distribution shows that marital status may have an impact on the variables being investigated, such as emotional intelligence and employee behavior. In terms of education, a considerable majority of respondents (64.2%) had a PhD or a professional certificate (22.6%), suggesting a well-educated sample group. This educational variety may lead to a more nuanced understanding of the relationships between politics, emotional intelligence, and employee behavior. In terms of income, the majority of respondents (35.0%) earn between Rs. 40,001 and Rs. 50,000 per month, closely followed by those earning Rs. 60,001 or more per month (34.0%). Finally, in terms of years of experience, a sizable majority of respondents had 2 to 5 years (43.0%), followed by those with 6 to 10 years (32.8%). This distribution indicates a somewhat experienced group, which may influence their perceptions and conduct at work.

The descriptive statistics presented in Table 2 (extended data) ( Sonakshi Bhatia *et al*., 2024b), illustrate the mean scores and standard deviations for various factors influencing organizational politics, emotional intelligence, and employee behavior within the studied context. For relationship conflict (RC), respondents reported a mean score of 3.5592 with a standard deviation of 0.92123, indicating moderate levels of friction and tension among employees, as well as frequent conflicts about ideas and work tasks. Similarly, role conflict (ROC) exhibited a mean of 3.4902 with a standard deviation of 0.84026, suggesting instances where individuals face challenges aligning with organizational roles and encountering conflicting demands. Resource constraints (REC) were perceived at a mean of 3.4575 with a standard deviation of 0.86520, indicating a moderate perception of insufficient resources hindering effective job performance. The need for power was reported with a mean score of 3.4828 and a standard deviation of.86388, reflecting perceptions regarding authority, access to information, and influence within the organizational hierarchy. Workforce diversity (WD) exhibited a higher mean of 3.7373 with a standard deviation of 1.00694, suggesting notable variations in values, goals, and objectives among employees. Perceived organizational politics (POP) displayed a mean score of 3.5230 with a relatively lower standard deviation of 0.73836, indicating moderate agreement towards political behaviors such as self-promotion and conformity to influential groups. Emotional intelligence (EI) was reported with a mean of 3.6010 and a standard deviation of 0.79514, reflecting moderate levels of self-awareness, emotional understanding, and emotional regulation among respondents. Lastly, employee behavioral dynamics (BD) demonstrated a mean score of 3.5390 with a standard deviation of 0.74679, suggesting varying degrees of intention to remain in the organization, task engagement, and energy exertion. These descriptive statistics provide insights into the perceived levels of organizational dynamics and individual attributes among the study participants.

### Factors influencing organizational politics, emotional intelligence, and employee behavior: a PLS-SEM model

**Measurement model**

In evaluating the measurement model for factors influencing organizational politics, emotional intelligence and employee behavior, rigorous attention to construct validity, reliability, and factorial validity is paramount. Ensuring the validity and reliability of measuring tools is critical for doing sound research in organizational psychology. First, construct validity is proven through theoretical foundation and empirical evidence ( Smith, 2018). This entails validating links between measured constructs and their theoretical frameworks. Second, reliability measures, such as internal consistency and test-retest reliability, are critical ( Jones *et al.*, 2020) High Cronbach's alpha values (0.805 to 0.972) show good internal consistency, whereas composite reliability estimates (rho_a and rho_c) greater than 0.7 (range from 0.812 to 0.975) confirm dependability ( Brown & Jones, 2019). Confirmatory factor analysis is used to measure goodness-of-fit indices like as chi-square, CFI, TLI, and RMSEA ( Brown *et al.*, 2021). Discriminant validity guarantees that the model differentiates across constructs; AVE values (0.567 to 0.836) greater than 0.5 indicate adequate convergent validity ( Black *et al.*, 2017). Overall, these data indicate that the measuring approach has strong reliability and validity, which increases the trustworthiness of the study findings ( Table 3).

**Table 3.** Construct reliability and validity.

	Cronbach's alpha	Composite reliability (rho_a)	Composite reliability (rho_c)	Average variance extracted (AVE)	Collinearity Matrix (VIF)
Behavioral Dynamics	0.972	0.972	0.975	0.799	
Emotional Intelligence	0.967	0.969	0.971	0.771	3.826
Need for Power	0.941	0.959	0.953	0.648	3.749
Perceived Organisational Politics	0.953	0.954	0.963	0.811	3.749
Relationship conflict	0.877	0.884	0.911	0.671	2.367
Resource Constraints	0.805	0.812	0.872	0.632	2.336
Role conflict	0.902	0.902	0.921	0.567	2.553
Work Force Diversity	0.902	0.902	0.938	0.836	2.553

Source: Author’s Compilation.

The Heterotrait-Monotrait Ratio (HTMT) is used to examine the discriminant validity of components, with values less than one indicating appropriate discriminant validity, as shown in Table 4. The HTMT matrix in this study depicts the relationships between various constructs such as Behavioral Dynamics, Emotional Intelligence, Need for Power, Perceived Organizational Politics, Relationship Conflict, Resource Constraints, Role Conflict, Workforce Diversity, and the interaction term Emotional Intelligence and Perceived Organizational Politics. Notably, all values in the diagonal (which represents a construct's connection with itself ) are more than 0.9, showing good convergent validity. Meanwhile, the off-diagonal components have values that are generally less than one, indicating discriminant validity between constructs. For example, the Emotional Intelligence and Perceived Organizational Politics interaction has the highest value of 0.34, suggesting the greatest association, although it is still below the threshold for discriminant validity. Overall, these findings support the distinctiveness of the constructs under investigation, crucial for ensuring the robustness of the study's theoretical framework.

**Table 4.** Discriminant validity: Heterotrait-monotrait ratio (HTMT) – Matrix.

	Behavioral Dynamics	Emotional Intelligence	Need for Power	Perceived Organisational Politics	Relationship Conflict	Resource Constraints	Role conflict	Work Force Diversity	Emotional Intelligence x Perceived Organisational Politics
Behavioral Dynamics									
Emotional Intelligence	0.951								
Need for Power	0.923	0.861							
Perceived Organisational Politics	1.006	0.889	0.920						
Relationship conflict	0.838	0.763	0.617	0.826					
Resource Constraints	0.847	0.730	0.806	0.853	0.460				
Role conflict	0.838	0.685	0.573	0.848	0.788	0.668			
Work Force Diversity	0.750	0.779	0.852	0.789	0.457	0.632	0.396		
Emotional Intelligence x Perceived Organisational_ Politics	0.258	0.287	0.286	0.251	0.249	0.103	0.223	0.340	

Source: Author’s Compilation.

The discriminant validity of the constructs was determined using the Fornell-Larcker criterion. The results reveal that all constructs have discriminant validity, since the square root of the Average Variance Extracted (AVE) for each construct (shown diagonally) exceeds the correlations between that construct and all other constructs. The diagonal elements reflect the AVE for each construct, but the off-diagonal elements show the correlations between them. For example, Emotional Intelligence has good discriminant validity (0.925), outperforming relationships with other dimensions such as Need for Power (0.878) and Perceived Organizational Politics (0.856). Similarly, additional constructs, such as Relationship Conflict and Resource Constraints, fit the requirement, with greater AVE values (0.819 and 0.795, respectively) than their correlations with other constructs. These findings suggest that the measurement model adequately discriminates between the constructs under examination, thus enhancing the robustness of the study's theoretical framework and contributing to the validity of the research instrument ( Table 5).

**Table 5.** Descriminnt Validity: Fornell-Larcker criterion.

	Behavioral Dynamics	Emotional Intelligence	Need for Power	Perceived Organisational Politics	Relationship conflict	Resource Constraints	Role conflict	Work Force Diversity
Behavioral Dynamics	0.894							
Emotional Intelligence	0.925	0.878						
Need for Power	0.886	0.830	0.805					
Perceived Organisational Politics	0.968	0.856	0.872	0.901				
Relationship conflict	0.779	0.708	0.560	0.760	0.819			
Resource Constraints	0.754	0.654	0.701	0.751	0.402	0.795		
Role conflict	0.790	0.647	0.532	0.792	0.712	0.572	0.753	
Work Force Diversity	0.703	0.730	0.778	0.732	0.406	0.541	0.366	0.914

Source: Author’s Compilation.

### Structural model and hypothesis testing

Table 6 shows the results of a regression analysis that looked at the relationship between several constructs, including Behavioral Dynamics, Emotional Intelligence, Need for Power, Perceived Organizational Politics, Relationship Conflict, Resource Constraints, Role Conflict, Workforce Diversity, and the interaction between Emotional Intelligence and Perceived Organizational Politics. The R-square values range from 0.733 to 0.972, indicating strong explanatory power across constructs. Furthermore, the f-square matrix demonstrates the relative relevance of each predictor in explaining variance within the constructs, with higher values suggesting greater influence. For example, Emotional Intelligence and Perceived Organizational Politics both contribute considerably to explaining variance in Behavioral Dynamics, as indicated by f-square values of 4.129 and 2.749, respectively. These findings indicate strong links between the researched dimensions, offering insights into organizational dynamics and suggested areas for further research.

**Table 6.** Structural Model and outcome: Path coefficients (Mean, STDEV, T values, p values).

	Original sample (O)	Standard deviation (STDEV)	T statistics (|O/STDEV|)	P values
Emotional Intelligence ->Behavioral Dynamics	0.361	0.018	20.227	0.000
Need for Power ->Perceived Organisational Politics	0.332	0.021	15.666	0.000
Perceived Organisational Politics ->Behavioral Dynamics	0.661	0.016	41.979	0.000
Perceived Organisational Politics -> Emotional Intelligence.	0.856	0.014	61.011	0.000
Relationship conflict ->Perceived Organisational Politics	0.220	0.015	14.261	0.000
Resource Constraints ->Perceived Organisational Politics	0.154	0.018	8.730	0.000
Role conflict ->Perceived Organisational Politics	0.300	0.017	17.754	0.000
Work Force Diversity ->Perceived Organisational Politics	0.191	0.017	11.339	0.000
Perceived Organisational Politics ->Emotional_Intelligence. ->Behavioral Dynamics	0.309	0.014	21.625	0.000

Source: Author’s Compilation.

In the structural model analysis, regression coefficients, t-values, and p-values were used to determine the correlations between different constructs. Path coefficients represent the intensity and direction of the associations. Notably, Emotional Intelligence significantly influenced Behavioural Dynamics (β = 0.361, t = 20.227, p < 0.001), while Need for Power notably affected Perceived Organisational Politics (β = 0.332, t = 15.666, p < 0.001). Perceived Organisational Politics was found to have substantial impacts on both Behavioural Dynamics (β = 0.661, t = 41.979, p < 0.001) and Emotional Intelligence (β = 0.856, t = 61.011, p < 0.001). Furthermore, different contextual elements such as relationship conflict, resource constraints, role conflict, and workforce diversity have a substantial impact on perceived organizational politics. A significant mediated association was found between perceived organizational politics, emotional intelligence, and behavioral dynamics (β = 0.309, t = 21.625, p < 0.001). Overall, all assumptions were confirmed, suggesting strong relationships between the components in the proposed model.

## Discussion

**Figure 1.**
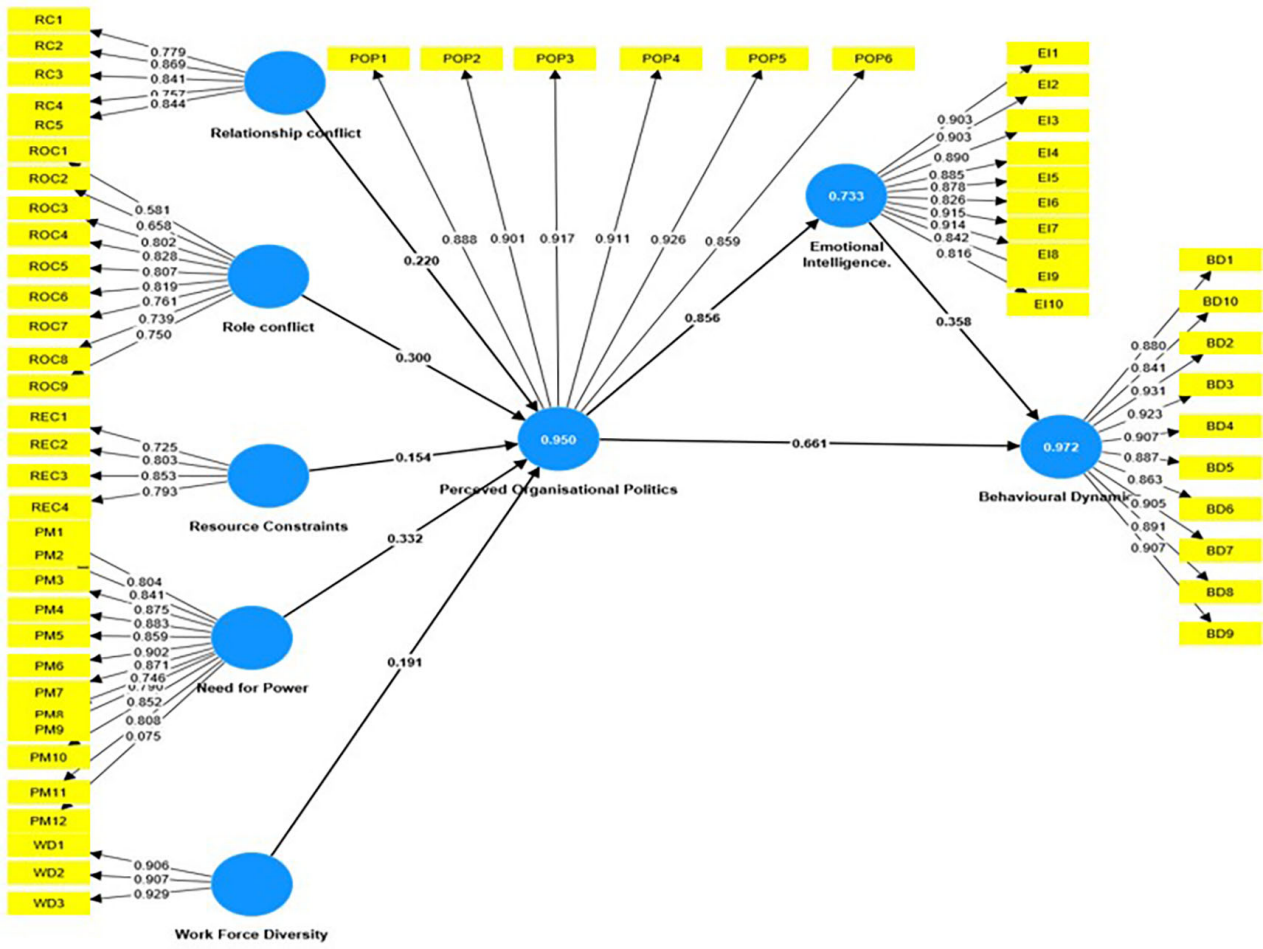
Mediation Analysis using SEM. Source: Author’s compilation.

Organizational politics, emotional intelligence, and employee behavior are all linked and have a significant impact on working conditions, as shown in the Figure 1. In a recent research that used Partial Least Squares Structural Equation Modeling (PLS-SEM), the interactions between these constructs were examined to identify their underlying dynamics. The findings found substantial connections, providing insight into the elements that influence organizational politics, emotional intelligence, and consequent behavioral consequences.

Emotional intelligence emerged as a critical element, having significant implications for organizational behavioral dynamics. This is consistent with previous studies demonstrating that persons with higher emotional intelligence display more flexibility, effective communication, and conflict resolution abilities, contributing favorably to overall workplace behavior ( Goleman, 1995).

Furthermore, the craving for power was found to be a strong predictor of perceived organizational politics. Previous research has confirmed this association, stressing how individuals motivated by a desire for power may engage in manipulative actions or strive to influence organizational dynamics to achieve their goals ( Kacmar & Ferris, 1991).

Perceived organizational politics, in turn, had a significant impact on emotional intelligence and behavior dynamics. This conclusion is consistent with previous research that has highlighted the negative consequences of perceived politics on employee attitudes, motivation, and interpersonal connections (D. L. Ferris *et al.*, 2008).

Furthermore, contextual factors such as interpersonal conflict, resource limitations, role conflict, and workforce diversity were found to have a substantial influence on views of organizational politics. Prior research has highlighted the influence of these contextual variables on organizational dynamics, stressing their significance in affecting employee attitudes and actions ( Jehn *et al*., 1997; Podsakoff *et al*., 2009; Jehn, 1995).

Furthermore, the study found a mediated link between perceived organizational politics, emotional intelligence, and behavioral dynamics. This emphasizes the intricate interplay between these dimensions, implying that organizational politics might act as a stimulant for the development of emotional intelligence and subsequent behavioral reactions.

Finally, the outcomes of this study illustrate the varied character of organizational dynamics, particularly the deep linkages between organizational politics, emotional intelligence, and employee behavior. Understanding these linkages allows firms to develop methods to limit the negative impacts of politics, encourage emotional intelligence, and promote good behavioral outcomes, resulting in increased overall organizational effectiveness and employee well-being.

The relationship between organizational politics and emotional intelligence is a generally neglected domain, despite its considerable impact on organizational behavior. Previous research indicates that individuals with elevated emotional intelligence exhibit superior regulation of their emotions and feelings, enabling them to manage stressful situations more judiciously, in contrast to those with lower emotional intelligence ( Abraham, 2000; Nikolaou & Tsaousis, 2002; Poon, 2003; Sy *et al.*, 2006). Our findings indicate that emotional intelligence influences perceptions of politics and indirectly impacts employees’ work attitudes and behaviors through a mediating effect on POP. This study not only contributes to the literature on organizational politics but also provides insights for the domain of emotional intelligence. Emotional intelligence is posited as a crucial element in alleviating the detrimental impacts of organizational politics. The notion of emotional intelligence (EI), which has garnered much attention in recent years, complicates this scenario by providing a possible safeguard against the frequently adverse effects of organizational politics. The relationship between organizational politics and emotional intelligence is a generally neglected domain, despite its considerable impact on organizational behavior. Previous research indicates that individuals with elevated emotional intelligence exhibit superior regulation of their emotions and feelings, enabling them to manage stressful situations more judiciously, in contrast to those with lower emotional intelligence ( Abraham, 2000; Nikolaou & Tsaousis, 2002; Poon, 2003; Sy *et al.*, 2006).

### Theoritical implication

The study adds theoretical value by revealing strong links between organizational politics, emotional intelligence, and employee behavior. Emotional intelligence has a significant impact on behavioral dynamics, whereas the desire for power shapes perceptions of corporate politics. The study also identifies contextual factors like relationship conflict, resource constraints, role conflict, and workforce diversity, which significantly impact perceived organizational politics. Moreover, it establishes a mediated relationship among perceived organizational politics, emotional intelligence, and behavioral dynamics. These findings offer valuable insights into understanding and managing workplace dynamics, contributing to the existing body of knowledge on organizational behavior. Implications for Practice: Understanding the interplay between organizational politics, emotional intelligence, and employee behavior has important implications for organizational leaders and practitioners.

### Managerial implication

The study “Factors Influencing Organizational Politics, Emotional Intelligence, and Employee Behavior: A PLS-SEM Model” underscores the managerial implications of its findings. It suggests that fostering emotional intelligence among employees can mitigate the negative effects of organizational politics on employee behavior. Managers should prioritize emotional intelligence development to cultivate a healthier work environment. The organizations should prioritize the development of emotionally intelligent leaders who can effectively manage the political dynamics within their teams and foster a culture of transparency and fairnessapart from this, organizations can implement training programs aimed at enhancing employees' emotional intelligence skills, such as self-awareness, self-regulation, social awareness, and relationship management. By equipping employees with the necessary emotional competencies, organizations can empower them to navigate organizational politics more effectively and respond constructively to challenging situations.

### Limitations of the study

One of the primary limitations of this study is the reliance on a specific sample size or demographic, which may limit the generalizability of the findings. The study's results may not be applicable to organizations with different structures, cultures, or demographics. Another methodologicla limitation of the study is chosing cross-sectional design of the study that restricts the ability to establish causal relationships between variables. Given that all data were collected through self-report measures, there exists a possibility of common method bias. Participants may have provided socially desirable responses or may have had difficulty accurately assessing their emotional intelligence or organizational politics behaviors. The study may not have accounted for all potential contextual factors that could influence the relationships under investigation. Factors such as organizational culture, leadership style, and industry-specific dynamics could moderate the relationships between organizational politics, emotional intelligence, and employee behavior.

### Future scope

Future study might look more into the mediating and regulating mechanisms that underpin the links between organizational politics, emotional intelligence, and employee behavior. Exploring elements like organizational justice, trust, or work satisfaction as potential mediators or moderators might help us better grasp the intricate interplay between these variables. It is proposed that future research be conducted on longitudinal studies, which might provide insights into the temporal dynamics of the correlations investigated in this work. Tracking changes in organizational politics, emotional intelligence, and employee behavior over time may give significant information regarding the long-term benefits and sustainability of interventions targeted at minimizing negative consequences related with organizational politics. Building on the study's findings, future research might create and assess intervention programs aimed at improving employees' emotional intelligence. Furthermore, training programs for organizational leaders should focus on ways for mitigating the detrimental influence of organizational politics on employee behavior and organizational outcomes. Finally, future study might look into developing trends, such as remote work arrangements or technology breakthroughs, and how these affect organizational politics, emotional intelligence, and employee behavior. Understanding how these changing dynamics influence workplace interactions and dynamics is critical for designing successful solutions to increase organisational performance and employee well-being in the digital age.

## Conclusions

This research digs into the complex interplay between organizational politics, emotional intelligence, and employee behavior, emphasizing their far-reaching ramifications in the workplace. Organizational politics may have a negative impact on employee attitudes, resulting in lower work satisfaction, higher turnover intentions, and poorer performance. However, emotional intelligence emerges as a critical mitigating element, allowing individuals to successfully traverse such circumstances. Organizations may equip staff to deal with political difficulties by cultivating emotionally intelligent leadership and a culture that values openness and justice ( Jordan & Troth, 2021). More study is needed to investigate additional elements that influence this link, providing deeper insights for building favorable workplace dynamics. The study found substantial links between emotional intelligence, the demand for power, perceived corporate politics, and employee behavior. Emotional intelligence has a favorable impact on behavioral dynamics, while the need for power correlates with perceived organizational politics ( Abraham, 2000). Additionally, contextual factors like relationship conflict and workforce diversity significantly impact perceived organizational politics.

### Ethics and consent

This research was conducted in accordance with guidelines of the **Research Ethics Board (REB)** of Uttaranchal University. The Research Ethics Board has given ethical approval and the approval number is UU/DRI/EC/2024/004. The formal ethical approval letter was obtained retrospectively. Prior to commencing our research, we obtained verbal approval from the Research Ethics Board (REB) of Uttaranchal University, allowing us to proceed with our study in the month of November 2023, as the REB of Uttaranchal University provides ethical approval in writing only when it is required by a journal. This preliminary verbal approval was based on our detailed proposal, which the REB reviewed and approved in principle, ensuring that all ethical standards and protocols were adhered to. Consequently, we initiated our research in good faith, strictly following the ethical guidelines outlined during the preliminary review. Our research was conducted with full ethical oversight and compliance from the REB from the outset.

The questionnaire has been submitted to REB of the university, the board members and chairperson have identified the viability of the research topic. All the members presented their research objectives to the board, then the questionnaire got approval to conduct the study.

### Consent statement

The written consent from all the participants involved in the study has been taken. A self-explanatory written statement was attached with the questionnaire for the participants and the similar questionnaire has been submitted to the university research board (REB).

## Data Availability

Figshare. Datasheet_Analyzing the Relationship Between Organizational Politics, Emotional Intelligence, and Employee Behavior A PLS-SEM Modeling, https://doi.org/10.6084/m9.figshare.25688832.v1 [ Sonakshi Bhatia *et al*., 2024a] Data is available under the terms of the CC BY 4.0 Figshare: Table 2 Factors Influencing Organizational Politics. https://doi.org/10.6084/m9.figshare.26500618.v1 [ Sonakshi Bhatia *et al*., 2024b] This project contains the following extended data:
•
Table 2 Factors Influencing Organizational Politics. Data is available under the terms of the CC BY 4.0
