# Supplementary material for: Factors Influencing Organisational Politics and their Relationship with Emotional Intelligence and Employee Behavior: A PLS-SEM Modelling
Source: F1000Res. 2025 Jun 2;13:996. Originally published 2024 Sep 2. [Version 4] doi: 10.12688/f1000research.151721.4 (PMC12123262; doi:10.12688/f1000research.151721.4)
Supplement: Peer review history

# Reviewer report: Reviewer response for version 4

**DOI:** 10.5256/f1000research.182820.r382408

**Published:** 2025-12-24

**Authors:** Zartashia Kynat Javaid

**Affiliations:** 1 Government College University Faisalabad, Faisalabad, Punjab, Pakistan

This study is a good contribution in workplace dynamics. There are some minor changes for improvement.

 Abstract lacks methodological details.i.e. sampling technique,  data collection 

 Introduction needs to be reorganized with clarity including theoretical underpinning here. 

 Add sample justification technique. Add some details of sample along with inclusion and exclusion criteria to select participants.

Is the work clearly and accurately presented and does it cite the current literature?

Yes

If applicable, is the statistical analysis and its interpretation appropriate?

Yes

Are all the source data underlying the results available to ensure full reproducibility?

No

Is the study design appropriate and is the work technically sound?

Yes

Are the conclusions drawn adequately supported by the results?

Partly

Are sufficient details of methods and analysis provided to allow replication by others?

Yes

Reviewer Expertise:

Psychology

I confirm that I have read this submission and believe that I have an appropriate level of expertise to confirm that it is of an acceptable scientific standard, however I have significant reservations, as outlined above.

# Reviewer report: Reviewer response for version 4

**DOI:** 10.5256/f1000research.182820.r389331

**Published:** 2025-06-06

**Authors:** Abílio Afonso Lourenço

**Affiliations:** 1 University of Minho, Braga, Portugal

I recommend this manuscript to be indexed. Approved.

Is the work clearly and accurately presented and does it cite the current literature?

Partly

If applicable, is the statistical analysis and its interpretation appropriate?

Partly

Are all the source data underlying the results available to ensure full reproducibility?

Partly

Is the study design appropriate and is the work technically sound?

Partly

Are the conclusions drawn adequately supported by the results?

Partly

Are sufficient details of methods and analysis provided to allow replication by others?

Partly

Reviewer Expertise:

Learning, Metacognition, Psychological Assessment, Self-Regulated Learning, Motivational Psychology, Conflict Studies, Teaching and Learning, Educational Psychology, Educational Assessment, Social Psychology, Educational Management, Assessment, Teacher Training, Teaching Methodology, Educational Evaluation, Technology Enhanced Learning, Student Development, psychometrics (SEM)

I confirm that I have read this submission and believe that I have an appropriate level of expertise to confirm that it is of an acceptable scientific standard.

# Reviewer report: Reviewer response for version 3

**DOI:** 10.5256/f1000research.182036.r386866

**Published:** 2025-05-30

**Authors:** Abílio Afonso Lourenço

**Affiliations:** 1 University of Minho, Braga, Portugal

Dear Author/s

 After a new reading of the article “Factors Influencing Organisational Politics and their Relationship with Emotional Intelligence and Employee Behavior: A PLS-SEM Modelling,” it is confirmed that it meets the conditions for indexing. This work not only contributes significantly to the academic literature but also holds the potential to influence practices and public policies, fostering a positive impact on human resource management, the promotion of healthier organisational environments, and the appreciation of emotional intelligence as a key factor for employee well-being and performance in complex organisational contexts.

 I reiterate my gratitude for the dedication shown throughout the process. I am confident that this article will be a valuable contribution to the field of study and inspire future research and discussions on this crucial topic.

 Kind regards,

 Reviewer for F1000Research

Is the work clearly and accurately presented and does it cite the current literature?

Partly

If applicable, is the statistical analysis and its interpretation appropriate?

Partly

Are all the source data underlying the results available to ensure full reproducibility?

Partly

Is the study design appropriate and is the work technically sound?

Partly

Are the conclusions drawn adequately supported by the results?

Partly

Are sufficient details of methods and analysis provided to allow replication by others?

Partly

Reviewer Expertise:

Learning, Metacognition, Psychological Assessment, Self-Regulated Learning, Motivational Psychology, Conflict Studies, Teaching and Learning, Educational Psychology, Educational Assessment, Social Psychology, Educational Management, Assessment, Teacher Training, Teaching Methodology, Educational Evaluation, Technology Enhanced Learning, Student Development, psychometrics (SEM)

I confirm that I have read this submission and believe that I have an appropriate level of expertise to confirm that it is of an acceptable scientific standard.

# Reviewer report: Reviewer response for version 3

**DOI:** 10.5256/f1000research.182036.r382413

**Published:** 2025-05-29

**Authors:** Angelos Ntalakos

**Affiliations:** 1 University of Thessaly, Volos, Greece

Regarding the methodology of the research. The authors should explain why they used the convenience sampling method. What about the response rate? Did all participants agree to answer?

 When constructing the Hypotheses the reader is a little bit confused about what are the basic variables of the research. For example workplace diversity is a sub - dimension of  organizational politics? The hypotheses should be more clear about the basic variables and their dimensions. 

 Moreover, hypotheses H1-H5 what kind of contribution they make on organizational politics? Positive, negative?

 In the results of the hypothesis testing it is not clear if the hypotheses were accepted or not and why.

Is the work clearly and accurately presented and does it cite the current literature?

Yes

If applicable, is the statistical analysis and its interpretation appropriate?

Partly

Are all the source data underlying the results available to ensure full reproducibility?

Yes

Is the study design appropriate and is the work technically sound?

Yes

Are the conclusions drawn adequately supported by the results?

Partly

Are sufficient details of methods and analysis provided to allow replication by others?

Yes

Reviewer Expertise:

Organizational Behavior, Leadership, Group Dynamics, Human Resources Management

I confirm that I have read this submission and believe that I have an appropriate level of expertise to confirm that it is of an acceptable scientific standard, however I have significant reservations, as outlined above.

# Reviewer report: Reviewer response for version 2

**DOI:** 10.5256/f1000research.179460.r382415

**Published:** 2025-05-23

**Authors:** Tibor Zsigmond

**Affiliations:** 1 J. Selye University, Komárno, Slovakia

Dear Authors,

 I see that the paper is the result of a hard work. I advise you the following:

 - there is a room for adding more new fresh studies. For e.g. Mura et al. (2021) stated: "It is worth employing an emotionally intelligent employee if the position requires teamwork or cooperation within and outside the company (e.g. corporate partners) or interaction with customers (customer service, in-store sales, other services, etc.)"

 - what is the min. age of the respondents. We see only "up to 25 years". The youngest respondents have to be over 18 years old.

 - I think the income level should be written in USD too. But I leave it on the Editor(s).

 - Table 4 + 5. should be on 1 page if possible. Also, it would be not hard to make these horizontally. You should make abbreviations for the columns and add the meanings at footnote under. It would make the reader experience much greater. E.g. writing "EI" instead of "Emotional Intelligence".

 - The order of the Tables and text should be rearranged too. 3 tables one after the other isn't looking great, while you mention Table 3 and 4 before the tables, and you mention Table 5 after them in the next paragraph.

 - Same appeals for Table 6. At (sub)chapter "Structural model and hypothesis testing" the order should be the following: 1) 1st paragraph 2) Table 6 and 3) paragraph "In the structural model analysis...".

 - The "Discussion" part needs to be stronger. You should mention more studies. The mentioned studies are quite old (from the beginning of 2000s). There were several studies about "EI" in the last 2 decades too.

Is the work clearly and accurately presented and does it cite the current literature?

Partly

If applicable, is the statistical analysis and its interpretation appropriate?

Yes

Are all the source data underlying the results available to ensure full reproducibility?

Yes

Is the study design appropriate and is the work technically sound?

Yes

Are the conclusions drawn adequately supported by the results?

No

Are sufficient details of methods and analysis provided to allow replication by others?

Yes

Reviewer Expertise:

Emotional intelligence, Human Resources, Human Resources Management, Employee Well-Being, Organizational Behavior

I confirm that I have read this submission and believe that I have an appropriate level of expertise to confirm that it is of an acceptable scientific standard, however I have significant reservations, as outlined above.

## References

[1] : The effects of emotional intelligence and ethics of SME employees on knowledge sharing in Central-European countries. *Oeconomia Copernicana* .2021;12(4) : 10.24136/oc.2021.030 907-934 10.24136/oc.2021.030

# Response

**Published:** 2025-05-24

**Authors:** Sonakshi Bhatia

In response to the reviewer’s insightful comments, I have made several enhancements to strengthen the academic rigor and clarity of the manuscript. To enrich the theoretical framework and ensure relevance, I have expanded the literature review and discussion sections by adding several recent research studies. I have also clarified that all the respondents were aged 20 years and above in the survey, ensuring ethical compliance and resolving ambiguity in the “up to 25 years” age category.

 The result section showing, Table 4 and 5 were reformatted horizontally with abbreviations and explanatory footnotes to enhance readability. Finally, I have substantially revised the discussion section by adding recent references, thus reinforcing the study’s relevance and aligning it with contemporary research in emotional intelligence.

# Reviewer report: Reviewer response for version 2

**DOI:** 10.5256/f1000research.179460.r376384

**Published:** 2025-04-29

**Authors:** Arjun Kumar Shrestha

**Affiliations:** 1 Kathmandu University School of Management, Lalitpur, Nepal

The authors have failed to address the comments given in the previous review. I did not find any significant improvement in the revised version. For example:

 1. Title not changed as suggested.

 2.  In the second paragraph of the introduction section the authors state that "This research examines the interaction between OP and EI". This is confusing as there are no hypotheses testing the interactive effect of OP and EI on employee behavior.

 3. No convincing rationale for conducting this study has been found in the revised version too.

 4. The introduction section does not give any hint about the first five hypotheses.  

 I would like to suggest the authors to go back to the comments given in the previous review and address them fully.

Is the work clearly and accurately presented and does it cite the current literature?

Partly

If applicable, is the statistical analysis and its interpretation appropriate?

Yes

Are all the source data underlying the results available to ensure full reproducibility?

Yes

Is the study design appropriate and is the work technically sound?

No

Are the conclusions drawn adequately supported by the results?

Partly

Are sufficient details of methods and analysis provided to allow replication by others?

Partly

Reviewer Expertise:

Organizational Politics, Workplace Spirituality, Emotional Intelligence, Organizational Behavior

I confirm that I have read this submission and believe that I have an appropriate level of expertise to state that I do not consider it to be of an acceptable scientific standard, for reasons outlined above.

# Reviewer report: Reviewer response for version 2

**DOI:** 10.5256/f1000research.179460.r376385

**Published:** 2025-04-08

**Authors:** Abílio Afonso Lourenço

**Affiliations:** 1 University of Minho, Braga, Portugal

I would like to thank the authors for incorporating the suggestions into the article "Analyzing the Relationship Between Organizational Politics, Emotional Intelligence, and Employee Behavior: A PLS-SEM Modeling", significantly enriching its content. The revisions underscore the importance of developing employees' emotional intelligence to mitigate the negative effects of perceived organizational politics and promote a positive work environment. I am confident that this study will make a valuable academic contribution.

 Best regards,

 The reviewer

Is the work clearly and accurately presented and does it cite the current literature?

Partly

If applicable, is the statistical analysis and its interpretation appropriate?

Partly

Are all the source data underlying the results available to ensure full reproducibility?

Partly

Is the study design appropriate and is the work technically sound?

Partly

Are the conclusions drawn adequately supported by the results?

Partly

Are sufficient details of methods and analysis provided to allow replication by others?

Partly

Reviewer Expertise:

Learning, Metacognition, Psychological Assessment, Self-Regulated Learning, Motivational Psychology, Conflict Studies, Teaching and Learning, Educational Psychology, Educational Assessment, Social Psychology, Educational Management, Assessment, Teacher Training, Teaching Methodology, Educational Evaluation, Technology Enhanced Learning, Student Development, psychometrics (SEM)

I confirm that I have read this submission and believe that I have an appropriate level of expertise to confirm that it is of an acceptable scientific standard.

# Reviewer report: Reviewer response for version 1

**DOI:** 10.5256/f1000research.166394.r355202

**Published:** 2025-01-16

**Authors:** Abílio Afonso Lourenço

**Affiliations:** 1 University of Minho, Braga, Portugal

**Previous note**

 I appreciate the opportunity to review this article, as it represents an excellent learning opportunity, deepening the understanding of the topic, stimulating critical reflection on the methodologies employed, and contributing to scientific advancement in this area.

 The article has great potential for publication, provided that substantial revisions are made to the original text. The topic is highly relevant, as it addresses how the dynamics of organizational politics, emotional intelligence, and employee behaviour directly influence the workplace environment, impacting well-being, motivation, and performance within organizations. Understanding these interactions is essential for fostering a healthier, fairer, and more productive culture, offering practical tools to improve interpersonal relationships and conflict management in professional daily life.

 The potential publication of this article would enable F1000Research to significantly strengthen the theoretical foundations of the topic under analysis. However, the study's conclusions must be properly clarified and substantiated, ensuring that the manuscript provides genuine value.

 The target audience for this work includes a diverse group of academic readers with varying levels of expertise, making it imperative that the content is presented, accessible, and in a well-structured manner. Maintaining F1000Research's high standards of quality in this area is fundamental. Below are suggested revisions to enrich the manuscript:

**Abstract**

 The abstract appears somewhat dense and lengthy, which may hinder its readability. Although it includes all the essential information, it could be reorganized and simplified to convey the ideas more clearly and concisely. This is partly due to an excess of methodological details, such as the specific mention of the region, the number of respondents, and the variables analyzed, which could be presented more succinctly. Additionally, some sentences are long and complex, packing in too much information, which makes quick comprehension challenging. There is an excessive focus on complementary elements, such as contextual factors (need for power, diversity, among others), which, while relevant, could be summarized. Moreover, there is some repetition of ideas, such as the influence of organizational politics and the impact of emotional intelligence, which are addressed more than once and could be handled more succinctly.

**Introduction**

 The introduction contextualizes the research topic but lacks a more direct connection to the detailed content of the abstract. It would be beneficial to briefly mention the analytical method used (PLS-SEM) and the study sample, which would better link the introduction to the specific approach of the investigation. Additionally, the introduction could highlight the main objectives of the article, going beyond the exploration of concepts to also focus on practical solutions for mitigating the negative effects of organizational politics. A quick reference to the key findings, such as the positive impact of emotional intelligence on behavioural dynamics and the significant influence of organizational politics, would make the text more comprehensive. Finally, it would be helpful to streamline the content, focusing on what the article truly addresses and leaving historical and theoretical details for the literature review, making the introduction more concise and aligned with the rest of the study.

**Review of related literature**

**Factors influencing organization politics**

 - To strengthen the foundation for the formulation of the hypotheses, it is suggested that certain aspects be addressed for each one.

**Relationship conflict:**

 - It is suggested to explore how relational conflict affects the workplace climate, creating tensions that encourage political behaviour, and to address the role of leadership and organizational culture, which can exacerbate interpersonal conflicts and foster political disputes. Introducing the idea of how factors such as emotional intelligence or conflict management policies help mitigate these effects, acting as mediators, would be beneficial. Presenting more recent studies or empirical evidence that reinforces the relationship between interpersonal conflicts and political behaviour in different contexts is also recommended. There is a significant amount of current literature on this topic.

**Role conflict:**

 - It is suggested to delve deeper into the subject by addressing the impact on employee well-being, as role conflict can lead to stress and burnout, making employees more likely to resort to political strategies to cope with pressure. A lack of clarity or poor communication from leaders can exacerbate conflict, creating opportunities for political dynamics to emerge. Organizational culture plays a significant role, as rigid structures or limited transparency can intensify conflicts and foster manipulative behaviour. Including practical examples of how employees handle these situations, such as forming alliances or manipulating information, could enhance the discussion. Proposing solutions, such as better defining responsibilities, providing emotional intelligence training, or reviewing internal processes, would add practical value. Updating the bibliography is also recommended.

**Resource constraints**

 - It can be suggested that resource scarcity, such as limited funding or staff, may harm productivity and work quality, prompting employees to resort to political strategies to achieve their goals. Furthermore, leadership decisions regarding resource allocation, when perceived as unfair, can exacerbate these behaviours. Another aspect is that in environments promoting competition over collaboration, political manoeuvring tends to increase (e.g., favouritism, lobbying, sabotage). Proposed solutions could include clearer resource allocation policies, transparent processes, and incentives for collaboration.

**Need for power**

 - It can be mentioned that employees with a strong need for power may create competitive and hostile work environments, hindering collaboration and negatively affecting team morale. On the other hand, leaders with this trait may manipulate processes or make biased decisions to strengthen their authority, thereby intensifying political games. Furthermore, understanding the internal motivations of these individuals, such as insecurity or the need for validation, can provide better insight into the phenomenon. The interaction between the need for power and factors like resource constraints or relational conflicts can further exacerbate organizational politics. Examples of this include information manipulation, exclusion of colleagues, or the formation of strategic alliances. To mitigate these effects, it is important to promote balanced leadership, provide structured feedback, and encourage a fairer distribution of power in the workplace. Presenting results from more recent studies would be beneficial.

**Workforce diversity**

 - The hypothesis could be more detailed by addressing different types of diversity, such as gender, age, culture, and educational background, and how each of these factors affects political dynamics in the workplace. It could be suggested that diversity may influence communication within the organization, as different communication styles and cultural perceptions can lead to misunderstandings and conflicts that fuel organizational politics. It could be explored how leaders need to adjust their styles to manage diverse teams, as an inclusive approach can reduce political behaviour and promote more collaboration. Including minority groups can foster a climate of trust and reduce tensions, helping to mitigate destructive political behaviour in the organization. Updating the bibliography would be beneficial.

**Organizational politics and employee behaviour**

 - To strengthen the idea, it would be interesting to explore how individual perceptions of organizational politics influence employee behaviour and performance, as well as the relationship between politics and emotional factors, such as stress, that impact productivity. Another interesting aspect would be to address the impact of organizational politics in modern contexts, such as remote work, and how organizational culture can amplify these effects. Ways to mitigate these impacts, such as clear communication and ethical training, could also be discussed.

**Organizational politics and emotional intelligence**

 - Emotional intelligence, being an important variable in the study, could be explored in terms of how it helps employees manage their emotional reactions to organizational politics, which facilitates adaptation and conflict resolution. It would be interesting to analyse how factors such as leadership style and organizational culture influence this interaction, as leadership that values emotional intelligence can mitigate the negative impacts of organizational politics. Another aspect would be to address in more detail how emotional intelligence can be trained and developed to improve the management of organizational politics, fostering a healthier and more collaborative environment.

**Emotional Intelligence and employee behaviour**

 - The hypotheses could be deepened by detailing how EI impacts specific behaviour, such as collaboration and problem-solving, and analyzing variations between roles or hierarchical levels. It would be useful to explore how organizational contexts, such as culture and sector, moderate this relationship, as well as consider cultural differences in the application of EI in diverse teams.

 Another point would be to discuss how EI can be developed through training and its impact on team dynamics. It may also be interesting to clarify the mechanisms through which EI mediates the relationship between organizational politics and behaviour, complementing recent studies to strengthen the hypotheses.

**Theoretical Framework**

 - Regarding EI, the study would be much more robust with the reference to the model and more recent studies by Mayer and Salovey. The authors provide a rigorous theoretical approach, strongly based on empirical research, being more focused on the academic field. They aim to understand EI as a specific form of intelligence and to create objective psychometrics to measure it. That is, more in line with the present study.

 In turn, Goleman made EI a widely known concept accessible to the general public, but with less scientific rigor compared to the models of Mayer and Salovey.

**Methods**

 This chapter should be structured differently and divided into subchapters to facilitate organization and reading. That is, separated into subchapters titled sample, procedures, instruments, and data analysis. This separation, in addition to aligning with best academic writing practices, would help present the methodological details clearly and systematically. As a suggestion:

 - The sample should state that convenience sampling was used, justified by the ease of access to participants from different sectors and organizational contexts. The sample size of 500 participants was determined to ensure an adequate level of demographic and organizational representativeness, including diversity in sectors, functions, and geographical location. The sample should be properly characterized concerning gender, age, organizational role, and other relevant aspects. It should be mentioned that participants were recruited through online platforms and professional networks, promoting broad coverage and diversity.

   - As for the procedures, it should be stated that data were collected in November 2023, following a cross-sectional study design suitable for the research objectives, as it allows the investigation of connections between variables at a single point in time. The process involved inviting participants, completing the scales, and ensuring confidentiality and ethics at all stages. It is important to mention that the entire procedure was carried out by the provisions of the Helsinki Declaration (2013) on studies involving human participants.

   - Regarding the instruments, it should be stated that the three scales used in the study are validated for the context in which the study takes place (India) and have proven validity and reliability in the literature. It should be mentioned how many factors each scale has, the total number of items, and the respective Cronbach’s alphas, as well as the range of the Likert scales used. Essential information should be presented to justify the validation of the scales. It should be highlighted that to validate the results obtained in the study, the scales must be validated for the Indian context. That is, psychometric scales must be validated for the context or country in which they are applied because cultural, linguistic, and social factors can influence how people interpret and respond to items. A scale developed in another country may not adequately capture the meanings or behaviour in the new context, compromising its validity (whether it measures what it intends to measure) and reliability (whether it produces consistent results). 

 Local validation ensures that the items are culturally relevant, semantically clear, and suitable for the target audience, avoiding distortions in results and allowing for accurate and scientifically robust comparisons.

 - In data analysis, it should be mentioned that the PLS-SEM model was used, which is suitable for complex models with latent variables and limited samples. The analysis was conducted in two stages: the assessment of the measurement model to verify the reliability and validity of the scales, and the assessment of the structural model to examine the relationships between variables. The *bootstrapping* method was applied to assess the significance of direct and indirect effects in the model. To interpret the data from the tables and figures, it is essential to indicate which types of analyses were used and which fit indices were considered for adjusting the PLS-SEM model. The respective minimum/maximum values and the authors supporting these values should also be provided.

**Results**

 Following the previous point, the "Results" chapter and its respective subchapters seem to contain information that should be, at least partially, in earlier sections such as "Sample", "Procedures", "Instruments", and "Data Analysis". To improve the organization and make the study clearer, I suggest restructuring the "Results" chapter as follows:

 First, demographic data such as age, gender, marital status, and education (presented in Table 1) should be included in the "Sample" section (this can be provided in detail), as they are crucial to describe the participant profile before analyzing the results. Then, the description of the psychometric scales used and the validation of constructs (including validity and reliability indices) should be placed in the "Instruments" section, as they are essential to justify the methodological robustness before presenting the findings.

 More technical elements, such as the measurement model, discriminant validity, factor analysis, and the use of "bootstrapping", belong in the "Data Analysis" part, as they help explain the methodological basis for interpreting the results. The analysis of the structural model and hypothesis testing (presented in Table 6) are correctly in the "Results" section, but they should be more separated from the descriptive and technical data to avoid confusion. This reorganization will make the study more fluid, clear, and free of redundancies.

**Factors influencing organizational politics, emotional intelligence, and employee behaviour: a PLS-SEM model**

**Measurement model**

**Structural model and hypothesis testing**

**Discussion**

 - The "Discussion" largely aligns with the study's objectives and can be improved with the restructuring suggested in the previous point. However, it could be clearer to highlight how the results directly align with the specific objectives of the study, such as exploring the influences of organizational politics on employees' attitudes and behaviour, mediated by emotional intelligence. For example, the description of how organizational politics directly influences behavioural dynamics and emotional intelligence could be more explicitly connected to the original hypotheses, to strengthen the relationship between the data and the study's objectives.

   - As previously mentioned, the bibliography should be updated to provide more robustness to the study's results.

**Theoretical implication**

 - The theoretical implications can be strengthened by exploring how the organizational environment, including politics, culture, and power dynamics, influences individuals' emotions, attitudes, and behaviour. This study contributes to organizational behaviour theory by highlighting how perceived organizational politics affect emotional intelligence and employee behaviour. These social exchanges complement this understanding, explaining how relationships in the workplace involve resource exchanges, and how a negative perception of organizational politics can harm trust and contribute to resistance behaviour and disengagement.

**Managerial implication**

 - To enrich this point, several aspects could be addressed. Managers can adopt strategies that reduce the negative perception of organizational politics, such as ensuring fair decision-making processes, communicating clearly, and establishing well-defined policies. It is also essential to invest in training to identify and manage interpersonal and role conflicts, reducing their impact on the work environment. Regular organizational climate assessments help adjust practices based on employee feedback. Encouraging collaboration, promoting diversity as a strategic resource, and implementing continuous feedback systems strengthen trust and reduce manipulative behaviour.

**Limitations of the study**

 - Other limitations that could be considered include the absence of longitudinal analyses, which would allow for the observation of how the relationships between organizational politics, emotional intelligence, and employee behaviour evolve. Additionally, the study did not explore the possible effects of additional mediating or moderating variables, such as trust climate or organizational support, which could deepen the understanding of the dynamics investigated. Another limitation is the lack of diversification in data sources; complementary data, such as evaluations from supervisors or colleagues, could reduce the self-report bias. Finally, the study did not account for cultural or contextual variations between different countries or regions.

**Future scope**

 - In addition to the suggestions mentioned, future studies could investigate how different leadership styles influence the relationship between organizational politics, emotional intelligence, and employee behaviour. Furthermore, interdisciplinary research could integrate approaches from positive psychology, exploring how mindfulness practices or well-being-focused interventions can strengthen emotional intelligence and mitigate the negative impacts of organizational politics. Another important aspect is using more diverse samples, including different sectors or global organizations, to enhance the generalizability of the results.

**Conclusions**

 - It is suggested to highlight the importance of sustainable interventions in developing emotional intelligence and reducing the negative perception of organizational politics so that the positive effects persist over time.

**References**

 - It is suggested that more references from studies from the last 5 years, including those from 2025, be included.

Is the work clearly and accurately presented and does it cite the current literature?

Partly

If applicable, is the statistical analysis and its interpretation appropriate?

Partly

Are all the source data underlying the results available to ensure full reproducibility?

Partly

Is the study design appropriate and is the work technically sound?

Partly

Are the conclusions drawn adequately supported by the results?

Partly

Are sufficient details of methods and analysis provided to allow replication by others?

Partly

Reviewer Expertise:

Learning, Metacognition, Psychological Assessment, Self-Regulated Learning, Motivational Psychology, Conflict Studies, Teaching and Learning, Educational Psychology, Educational Assessment, Social Psychology, Educational Management, Assessment, Teacher Training, Teaching Methodology, Educational Evaluation, Technology Enhanced Learning, Student Development, psychometrics (SEM)

I confirm that I have read this submission and believe that I have an appropriate level of expertise to confirm that it is of an acceptable scientific standard, however I have significant reservations, as outlined above.

# Response

**Published:** 2025-03-14

**Authors:** Sonakshi Bhatia

I have made the changes as per the reviewer. i have updated the literature review , refined the gaps, and elaborated the discussion section to clear the concepts and rationale of the study.

 I have updated the latest literature reviews and updated the refernces also.

# Reviewer report: Reviewer response for version 1

**DOI:** 10.5256/f1000research.166394.r340212

**Published:** 2024-12-02

**Authors:** Arjun Kumar Shrestha

**Affiliations:** 1 Kathmandu University School of Management, Lalitpur, Nepal

The authors have chosen a pertinent issue for their study. They need to work on the following areas to further enhance the quality and relevance of the paper.

**Title:**

 From the first five hypotheses, it is evident  that one of the objectives is to identify the antecedents of POP, but it is not reflected in the title.

**Introduction:**

 This sections fails to succinctly convey the readers the need for conducting this study.  Meisler and Vioda-Gadot (2014) [Ref 1] have tested the same model in their study. Several other scholars have also tested similar model in the past. The authors need to provide a strong rationale for conducting this study and the contributions of the study in the introduction section.  

**Literature Review:**

 The authors need to add more recent literature in this section. More importantly, the theoretical support, i.e., which theory or theories help establish the proposed hypotheses and how should be clearly explained. The empirical supports and arguments made by the authors for H7 (Organizational politics have significant effect on employee emotional intelligence) are contradictory. First, the authors conclude, " ..... literature emphasizes the necessity of addressing organizationals politics as a major element impacting employees emotional intelligence ...". After this, citing past literature, they suggest that emotional acts as a buffer on the relationship between perceived organizationals politics and employee attitudes and behaviors. In this case, emotional intelligence acts as a moderator, not the antecedent of perceptions of organizational politics.

**Methods:**

 The authors are required to provide the details of all the scales used for measuring the study constructs. The authors have not stated which scales they used for measuring the antecedents of perceptions of organizational politics. For measuring perceptions of organizational politics, they have used Kacmar and Ferris's (1991) [Ref 2] scale. This scale is a three dimensional scale with 12 items. Similarly,  Wong and Law's  emotional intelligence scale is a multi-dimensional scale with four dimensions having four items in each dimension. However, in the analysis (Figure 1), the authors have treated both the scales as unidimensional scales. The number of items are also six and 10 respectively. The authors need to provide clear explanation for these discrepancies. They are also required to provide the details regarding the scales, i.e., the number of items in the scale, the authors of the scale, and the scoring format/anchors etc.

**Results:**

 Appropriate statistical tools have been used for testing hypotheses. 

**Discussion:**

 The authors found that emotional intelligence mediates the influence of perceived organizational politics on behavioral dynamics. Previous studies, e.g., Meisler and Vioda-Gadot's (2014) study, on the other hand suggested that perceptions of organizational politics mediates the relationship between emotional intelligence and employee attitudes and behaviors. The authors need to discuss these findings in detail.

**Language:**

 Editing for clarity, coherence, and grammatical accuracy is necessary. For example, the tense in the methods section, “Participants will be recruited,” should be revised to past tense.

**Citations:**

 Inconsistent citation style should be corrected.

Is the work clearly and accurately presented and does it cite the current literature?

Partly

If applicable, is the statistical analysis and its interpretation appropriate?

Yes

Are all the source data underlying the results available to ensure full reproducibility?

Yes

Is the study design appropriate and is the work technically sound?

No

Are the conclusions drawn adequately supported by the results?

Partly

Are sufficient details of methods and analysis provided to allow replication by others?

Partly

Reviewer Expertise:

Organizational Politics, Workplace Spirituality, Emotional Intelligence, Organizational Behavior

I confirm that I have read this submission and believe that I have an appropriate level of expertise to confirm that it is of an acceptable scientific standard, however I have significant reservations, as outlined above.

## References

[1] : Perceived organizational politics, emotional intelligence and work outcomes. *Personnel Review* .2014;43(1) : 10.1108/PR-02-2012-0040 116-135 10.1108/PR-02-2012-0040

[2] : Perceptions of Organizational Politics Scale (POPS): Development and Construct Validation. *Educational and Psychological Measurement* .1991;51(1) : 10.1177/0013164491511019 193-205 10.1177/0013164491511019

# Response

**Published:** 2025-03-14

**Authors:** Sonakshi Bhatia

We have revised the introduction section to provide a stronger rationale, explicitly outlining how our study builds upon and extends previous research. We have thoroughly edited the manuscript to correct tense inconsistencies, including revising statements like “Participants will be recruited” to the past tense. We have carefully reviewed and standardized the citation format throughout the manuscript to ensure adherence to a consistent referencing style.

 I have revised the hypothesis and cleared the scales used in the RM part. I have cleared the gaps and rationale of the study and enhanced the discussions section also. I have updated the references and updated.
